# Intervention strategies to promote healthy and sustainable food choices among parents with lower and higher socioeconomic status

**DOI:** 10.1186/s12889-022-14817-y

**Published:** 2022-12-19

**Authors:** Marjolijn Vos, Benedicte Deforche, Anneleen Van Kerckhove, Nathalie Michels, Maggie Geuens, Wendy Van Lippevelde

**Affiliations:** 1 Department of Marketing, Innovation and Organization, Faculty of Economics and Business Administration, Ghent University, Ghent, Belgium; 2 Department of Public Health and Primary Care, Faculty of Medicine and Health Sciences, Ghent University, Ghent, Belgium; 3 Movement and Nutrition for Health and Performance Research Group, Faculty of Physical Education and Physiotherapy, Vrije Universiteit Brussel, Ixelles, Belgium

**Keywords:** Interventions, Meal boxes, e-health, Nudges, Family, Socioeconomic position

## Abstract

**Background:**

A global shift towards more healthy and sustainable diets is necessary for the prevention of obesity and chronic diseases, as well as for the growing pressure on our ecosystems. Given that parents are important actors in affecting dietary behaviors of their children, developing intervention strategies targeting families and their practices is promising to reach positive behavior change among children. Also, it is important to tailor these interventions to the needs of parents with different socioeconomic statuses (SES), given that health inequalities continue to grow. This study aims to investigate perspectives of lower and higher SES parents on the usability and acceptability of various innovative intervention strategies.

**Methods:**

Fourteen focus groups and four individual interviews (*n* = 78, n_lowerSES_ = 17; n_higherSES_ = 61) were conducted in Belgium. A semi-structured interview guide was used to facilitate the discussions. The interviews were recorded, transcribed, and analyzed via thematic content analysis using NVivo.

**Results:**

To encourage healthy and sustainable food choices, interventions via online food shopping platforms and nudging strategies in grocery stores were mostly cited by higher SES parents, but these were less applicable for lower SES parents as they buy less online and mainly consider the price of products. Mobile applications that provide inspiration for healthy and sustainable recipes and easily accessible shopping lists received moderate support among lower and higher SES parents. Furthermore, both lower and higher SES parents showed interest in meal boxes delivered at home, but lower SES parents have not yet tried such meal boxes because of their higher prices. Still, both groups of SES parents mentioned many advantages of these meal boxes, such as the convenience and time-saving component, as well as the cooking inspiration aspect.

**Conclusion:**

Our study reveals the preferences of lower and higher SES parents for practical intervention strategies, providing insight in what features these strategies should have to be acceptable and useful. Hence, the findings can inform the development of a tailored family-based intervention strategy to improve parental food choices in favor of increased health and sustainability.

## Introduction

Encouraging people to adopt a healthy and sustainable lifestyle is a necessary challenge to tackle the rise of chronic diet-related noncommunicable diseases [1], while also reducing greenhouse gas emissions [2]. Obesity is associated with serious medical and psychosocial risks [3]. According to the World Health Organization, globally 39% of adults and 18% of children aged 5–19 suffer from overweight, and 13% of adults and 7% of children have obesity [4]. Furthermore, current dietary patterns negatively affect the natural environment and contribute to global warming, which may in turn exacerbate poor health [5]. Hence, improving dietary habits is crucial to improve health and reduce environmental impacts. According to the EAT-Lancet Commission, a universal diet that meets both these goals (i.e., a “win–win diet”) would mainly be based on plant-based foods and to a lesser extent on animal-based foods [5]. Indeed, replacing animal-based foods with plant-based foods is not only associated with reduced overall mortality [6], it has the strongest positive impact on environmental footprints [2, 7].

Given that food preferences and eating habits are often established in childhood and track into adulthood [8–10], research on the best approach to ameliorate children’s diet is crucial. Parents are key actors in reaching children, since food parenting practices (i.e., food availability, role modeling and family meal routines at home) have a strong influence on children’s food consumption [11, 12]. In turn, parenting practices are affected by parents’ food choices, making it a promising behavior to target with an intervention strategy. Herein, it is important to include parents with varying socioeconomic status (SES), since there is a social gradient in health and diet, already starting in younger ages [13, 14]. Not only do parents of lower SES model less healthy eating behaviors to their children than their higher SES counterparts [15], lower SES is also associated with lower diet quality [16, 17]. In economically advanced countries socioeconomic inequalities in overweight and obesity are even increasing [18]. These inequalities in healthy eating are complex, and driven by multiple factors such as psychological, social and economic determinants [15]. For example the affordability of foods is both influenced by the household income as well as the cost of healthy foods [16, 17].

It has been shown that health promoting interventions have a different impact on lower and higher SES groups, with purely informative interventions being less effective for lower SES groups, possibly even widening inequalities compared to more structural interventions that make changes in the environment [19–21]. According to Adams and colleagues [22], the effectiveness of interventions depends on the amount of “agency” (i.e., personal resources) individuals must use to benefit from the intervention. People with lower SES tend to have not only less financial means, but also less psychological resources, for example to resist children’s pester power for unhealthy foods [23], as well as less cognitive resources [24]. The latter are less needed in structural interventions like taxes, subsidies and altering portion sizes (a so-called “nudge”) [22]. To reach both lower and higher SES groups, it is therefore important to not only use informative interventions but also structural ones [22, 25]. Moreover, an effective implementation of an intervention strategy depends on its perceived acceptability [26]. However, limited research exists on the acceptability and usability of different interventions to promote healthy and sustainable food choices in both lower and higher SES parents. This qualitative study therefore aims to understand what interventions both parent groups prefer and find useful and acceptable. The study builds further on the paper by Vos et al. [27], where individual and environmental determinants of parental food choices were investigated. The usefulness and acceptability of intervention strategies is closely linked with the different reasons why parents buy or not buy healthy and sustainable foods.

## Method

### Study design

Fourteen focus groups and four individual interviews using a semi-structured interview guide were conducted in Belgium between March 2020 and May 2021. Focus groups stimulate an interactive discussion and allow for a deeper comprehension of what intervention strategies parents of varying SES prefer and why [28]. Also, individual interviews were carried out because of the difficulty to meet with a group of people during restrictive Covid-19 measures.

### Participants and recruitment

Only parents who were sufficiently proficient in Dutch or English and who had at least one child aged six to twelve years were eligible for participation. The upper limit for the child(ren)’s age was set at twelve because at this age children become adolescents and gain more behavioral autonomy and decision-making power regarding dietary choices [29]. Purposive and convenience sampling via social media was used to select higher SES parents. Lower SES parents were recruited via purposive sampling, by contacting and visiting social organizations (i.e., social restaurants, community health centers) and by handing out flyers. In some cases, a contact person from within the social organization who worked with the lower SES group helped recruiting participants. This way also two parents with a younger child of four years old were accidentally included in two focus groups, and included in the analysis.

In total, 78 parents participated in the study, with nine focus groups and one interview among higher SES, and five focus groups and three interviews among lower SES (see Table 1). Due to the Covid-19 outbreak and the following lockdown, planned data collection had to be revised. First, higher SES parents were interviewed in online focus groups via ZOOM. When Covid-19 rules became a little less strict, it was possible to conduct four focus groups and one interview with lower SES groups in a real life setting, while two other interviews again had to be held online. To promote participation of lower SES parents, a gift voucher was offered in three focus groups (i.e., for a total of 10 participants). Despite recruiting around six participants per focus group session, no-show rates were noticeably high, explaining for the smaller numbers of participants in the lower SES groups.

**Table 1:** Overview of focus groups and interviews

	**Date**	**Online/Offline**	**Number of participants**	**SES: High/Low**
**1**	1 March 2020	Offline	7	High
**2**	14 March 2020	Offline	5	Low
**3**	30 March 2020	Online	6	High
**4**	7 April 2020	Online	7	High
**5**	8 April 2020	Online	5	High
**6**	14 April 2020	Online	8	High
**7**	15 April 2020	Online	6	High
**8**	16 April 2020	Online	4	High
**9**	4 May 2020	Online	6	High
**10**	22 June 2020	Online	6	High
**11**	13 August 2020	Offline	1	Low
**12**	20 August 2020	Online	3	Low
**13**	28 September 2020	Offline	3	Low
**14**	2 October 2020	Offline	2	Low
**15**	12 October 2020	Offline	6	Low
**16**	20 May 2021	Online	1	Low
**17**	25 May 2021	Online	1	Low
**18**	8 June 2021	Online	1	High

### Data collection

Data collection was conducted by the first author (MV) and five research assistants. The whole data collection process was monitored and coordinated by the first author. The research assistants received a training and a written guide on how to conduct focus groups. The focus groups and interviews lasted at maximum one hour and were audiotaped. Prior to participation, participants signed an informed consent form and filled out a sociodemographic survey. The semi-structured interview guide consisted of two parts. First, questions about healthy food choices were asked, after which the same questions for sustainable food choices were addressed. To describe healthy and sustainable diets to participants, we followed the Flemish guidelines of the “food pyramid” [30]. Main recommendations for both healthy and sustainable diets are to eat plant-based foods as a basis and reduce consumption of animal-based foods. For a sustainable diet, also seasonal and local products are recommended while an additional recommendation for healthy diets is to limit the consumption of products with high sugar, salt, and fat content.

Being part of a larger study, the interview guide included questions on determinants of food choices (these results, the sociodemographic survey, and the semi-structured interview guide are published in Vos et al. [27]). This paper, however, focuses on reporting parents’ preferences, usability and acceptability of practical intervention strategies to promote healthy and sustainable diets. Following an introduction and warm-up exercise, discussions started with the question: *“What does healthy/sustainable food mean to you?”.* After providing insight into the definition and finding out what individual and environmental determinants were, the discussions continued with another open question: “*What could help you to make a more healthy/sustainable food choice?”*. Participants could generate ideas freely, and if no more ideas came to mind, their thoughts on three main interventions were asked: mHealth interventions (e.g., via applications and online shopping), meal boxes and grocery store nudges (e.g., labels, store layout). Prior research suggests these are promising interventions to influence food selection [28, 31–37].

### Data analysis

The focus group discussions and interviews were transcribed and analyzed using NVivo. Based on an iterative process, data collection was alternated with data analysis and reflection. Based on the thematic analysis method by Braun & Clarke [38], deductive (predetermined interventions listed above) and inductive reasoning (generation of other interventions) helped generating themes and analyzing data [38]. Working together with research assistants made it possible to achieve researcher triangulation. Five research assistants conducted the first 11 focus groups (eight with higher SES and two with lower SES parents) and one interview (with a lower SES parent) and analyzed them, a task that was repeated by the first author (MV). Moreover, the first author read each transcript and gave feedback on the interview process, making it possible to adjust the interview guide along the way. One extra focus group and one interview with higher SES parents was conducted by the first author, confirming that data saturation was reached. All five focus groups and one interview among lower SES parents were individually analyzed by the first and last author (WVL). Moreover, to find out if data saturation was reached, the first author conducted two extra online individual interviews with lower SES parents.

Demographic analysis was performed using SPSS Statistics 26. A Pearson’s chi-square test and Fisher exact test with *p* < 0.05 significance was used to compare demographics between lower and higher SES parents. Parents were categorized in lower or higher SES based on the recruitment place (social media vs. social organizations). To verify group assignment, a SES score for each participant was calculated based on income, educational degree, and profession (following Reynders et al. [39]). A participant was classified as lower SES if the index score was below 3. 76 parents were categorized correctly, two were in the wrong group; One parent participated in a focus group of lower SES but the index score indicated higher SES, and the same happened for a parent of lower SES who was recruited for an interview. As a result, this interview was analyzed as higher SES. For details about the calculation of the index score, see Table 2.

**Table 2:** Index score (Reynders, Nicaise & Van Damme, 2005)

**Variable**	**Category score**
Netto monthly family income	1. < 1000 euro 2. 1000–2000 euro 3. 2000–3000 euro 4. 3000–4000 euro 5. 4000–5000 euro 6. > 5000 euro
Parents’ highest degree	1. No education / primary education 2. Lower secondary education 3. Higher secondary education 4. Higher education 5. University
Parents’ profession	1. Never had a profession/ no profession 2. Uneducated blue collar employee 3. Educated blue collar employee 4. Farmer / self-employed small business 5. White collar employee / teaching assignment lower and secondary education 6. White collar employee management / teaching assignment higher education and university 7. Board member /liberal profession / self-employed medium sized business
**Formula to calculate the index score:** Mean (monthly income + mean (category score_degree parent 1 + 2) + mean (category score_profession parent 1 + 2))	

## Results

### Demographics

In total, 78 participants were involved in the focus groups (90% female and 10% male). The mean age in the higher SES group was 40.5 ± 4.8 years, and in the lower SES groups 37.9 ± 10.3 years. The higher SES participants were almost exclusively of Belgian origin (93%), while only 33.3% of the lower SES participants were of Belgian origin. Only 7.1% of the higher SES parents did not have a partner, whereas in the lower SES group 38% was single or divorced. Other significant differences between the two groups were found in the categories education, profession, and monthly income. Where most higher SES parents had a university (21%) or higher education degree (58%), most lower SES parents had a secondary education degree (40%). Regarding profession, higher SES parents often worked as white collar employee (77%), while most lower SES parents did not have a profession (71%). Family net monthly income was generally situated in the higher categories (> €3000) for higher SES parents (91%), and in the lower categories (< €1000, €1000–2000) for lower SES parents (86%). A complete overview of the demographics can be found in Vos et al.[27].

### Intervention strategies for healthy and sustainable food choices

As described in the methods section, we asked participants’ ideas about what could help to make a more healthy and sustainable food choice. Many higher SES parents spontaneously generated ideas. For sustainable food choices, higher SES parents cited that the media could help to raise awareness, and that a clear label and visualization in the grocery store, an application to increase knowledge, and a cheaper price for sustainable products, were valued. For healthy food choices, higher SES parents mentioned the Nutri-Score label, a seasonal vegetable and fruit calendar, meal boxes for variation and less temptations because of a lower frequency of store visits, fruit and vegetable packages from local farmers, and mobile applications (i.e., from a supermarket chain and a famous cook who offer recipes and grocery lists). Lower SES parents needed more probes and examples to answer this question, however, one parent spontaneously mentioned that a mobile application with more information on sustainable foods would be educational. In what follows, all results (i.e., answers on the open-ended question, as well as on the more specific strategy-directed questions) are presented according to each theme or intervention strategy, explaining possible differences in lower and higher SES parents and specifying distinctions for healthy and sustainable food choices when these were clearly mentioned.

#### Nudging

Many higher SES parents mentioned visibility enhancements as a helpful strategy for sustainable and healthy food choices. They preferred healthy products being placed at the cash register, at eye level or making them stand out more. Also reorganizing the store so fruit and vegetable departments are the first departments encountered, was indicated helpful by many higher SES parents as well as by one lower SES parent. Similarly, some higher SES parents thought it would be beneficial for sustainable food choices if Belgian or local products were put together. Nevertheless, a few other higher SES parents felt visibility would not affect them because they strictly stick to their shopping list. Also, convenience enhancements were mentioned as useful and acceptable for product choice by most of the higher SES parents, such as creating a supply of healthy and/or sustainable and pre-cooked meals, offering more variation in healthy and/or sustainable product alternatives, and increasing the relative share of healthy and sustainable alternatives in product assortments. Lower SES parents only mentioned paying attention to price promotions in grocery stores. A few higher and lower SES parents discussed hedonic interventions to influence healthy food choices, such as tasting sessions. Some higher SES parents were interested in healthy tasting sessions, possibly in combination with a recipe.

*“I find it convenient what is in store here (…) Brussels sprouts that are already prepared. When you don’t have much time, you just have to put it in the microwave.” [P*_*high*_* F, 44 yrs]*

*“I really like it when they put the vegetables and fruits in the beginning of the store, and before the corona there was a box with some fruit for children. That was really a really nice thing.” [P*_*low*_* F, 26yrs]*

Evaluative nutritional labels were mentioned by most higher SES participants. The Nutri-Score [40] is a popular label for healthy food choices, and was mostly perceived useful in providing information and raising awareness for themselves as well as for their children. Also for sustainable food choices, some higher SES parents indicated that a label like the Nutri-Score would be helpful. Disadvantages of evaluative nutritional labels were the ambiguity, the lack of confidence in the correctness of the label and an abundance of different labels. In addition, some parents found it time consuming to check labels while shopping. Therefore, parents proposed two comprehensive uniform labels, one for healthy products (such as the Nutri-Score) and one for sustainable products. Only a few lower SES parents mentioned the Nutri-score as a useful strategy. Some lower SES parents never heard of it but showed interest to find out more about the label. Most lower SES parents though were not interested in labels for healthy and sustainable food choices because they like to buy what they want without checking a label. And just like higher SES parents, some lower SES parents mentioned not trusting labels and finding them time consuming to read. Also descriptive nutritional labeling could encourage healthy food choices, as was mentioned by a few lower SES parents. They preferred looking at the separate ingredients, like how much sugar the product contains.

*“You have to shop for 5 people, it takes time, you already have a big shopping list, I don’t look at labels. I just take what I need.” [P*_*high*_* F, 48yrs]*

*“I think it would influence me, (…) if a product would show that it is more sustainable, and the price difference is reasonable, then I would undoubtedly go for the sustainable choice.” [P*_*high*_* F, 34yrs]*

*“I will still always look at the price, and organic foods are not really cheaper.” [P*_*low*_* F, 39yrs]*

#### Intervention strategies via online grocery shopping platforms

Higher SES parents indicated to increasingly shop for groceries online. Parents liked the user-friendliness, the fact that there is less temptation to buy unnecessary products, and the time-saving aspect. However, other higher SES parents still preferred shopping in the grocery store because they enjoyed it more, and one parent mentioned buying more varied fruits and vegetables when shopping in-store. Parents gave suggestions for improvement of existing online stores: they should include meal schedules, a shopping list and the ability to directly add ingredients from recipes to their shopping list. An added value would be if products were labelled or scored based on healthiness (i.e., the Nutri-Score) and sustainability (e.g., travel distance, origin). In contrast, lower SES parents did not indicate shopping for groceries online. They indicated to have doubts about the product's quality, not being interested in shopping online or having enough time to go to the store. One lower SES parent would be interested to shop online if there was an option to buy all the ingredients of a recipe in one click. However, this recipe should be culturally approved by the family.

*“It would be nice that when you click on a product, it would say ‘you can make this with it’, and you can find nutritional information.” [P*_*high*_* F, 42yrs]*

*“No, I prefer to walk in the store. Then I sometimes look at the price, the red promo price. That is interesting. Otherwise you don’t see it.” [Plow F, 48yrs]*

#### Mobile applications

Possibly mobile applications (apps) are an appropriate way to support parents’ healthy and sustainable food choices. Many higher SES parents preferred one comprehensive application that is convenient to use (e.g., scanning products and viewing the healthiness or sustainability of the products at a glance in the shop). Apps should feature a shopping list, a filter function for healthy and sustainable foods, recipes with ingredients and a possibility to order products online. Also apps should be free of charge, time-saving, and fulfill an informative function by providing information about nutrients and alternative products. A few parents suggested an option to deliver products at home. Nevertheless, half of the higher SES participants indicated that they were not interested in using apps, because they were found to be annoying, too time-consuming, or not appropriate for their age. Also, some parents preferred the whole shopping experience over watching their mobile screens when food shopping in the supermarket. When asking lower SES participants, similar results for healthy and sustainable food choices were found: parents were mostly interested in an app that provides a week menu, that saves time when shopping, that is easy to use, offers a filter function with alternative products (i.e., healthier or more sustainable), composes meal schedules, and provides inspiration via recipes. However, half of the lower SES parents were not interested in using an app, and expressed the importance of the price of foods.

*“Yes, an app would be interesting, to find variation and inspiration.” [P*_*low*_* F, 29yrs]*

*“I would be happy with this app, if it was a week menu coupled with a grocery list (…) to reduce my choice stress, yes.” [P*_*high*_* F, 48yrs]*

#### Meal boxes and other food packages

Meal boxes were considered a helpful strategy to make healthy and sustainable food choices by higher SES parents. Reasons were related to the convenience of an all-in-one package (i.e., time-saving and easy to order online), and the reduced temptation because of less frequent store visits. Furthermore, parents liked the meal boxes because of the range of available meal box options (e.g., vegan, meat, child friendly, fish), meal inspiration, nutrition information and the ability to learn new cooking skills. The meal boxes were perceived as healthy and varied. Choosing meal boxes for sustainable reasons is linked to less food waste, seasonal vegetables and a vegan or vegetarian option. Higher SES parents suggested that meal box providers also include more information about the sustainability of products. Furthermore, some parents stated that it would be helpful if grocery stores provided pre-packed healthy and/or sustainable food packages with all ingredients for a recipe. However, not all higher SES parents were in favor of meal boxes. Some parents pointed out difficulties in child friendliness of meal boxes, namely that the children do not like it and other dishes have to be prepared. Furthermore, the high price of meal boxes was mentioned, as well as doubts about sustainability of the boxes due to the amount of packaging waste and the transportation for home delivery. Even though most participants found meal boxes time saving, for some the preparation of recipes was rather time consuming. Also, a few parents did not like the pre-determined menus and having to cook it when they feel like eating something else that day. Lower SES parents mostly mentioned the expensiveness of the box as a barrier. If the meal boxes would be cheaper, some parents indicated wanting to use them because of the time-saving aspect, the convenience and to receive new inspiration (e.g., for vegetarian meals). However, a few parents preferred choosing their own products and recipes, also because of the risk of food waste if their children would not like it.

*“But in busy periods, for me it was a real convenience. Because everything was there, and I knew, that evening I just had to take it and cook it.” [P*_*high*_* F, 42yrs]*

*“There are things they do not like (children), and moreover it is always so much work, (…) it was not fixing something up quickly.” [P*_*high*_* F, 43yrs]*

*“Yes, if the price is good, I will order because it is good to receive a box with healthy food.” [P*_*low*_* F, 38yrs]*

*Interviewer: “Would you like to receive a meal box?” Participant: “No no. For the children. I think then that I do not like it.” [P*_*low*_* F, age unknown]*

Furthermore, fruit and vegetable packages were mentioned by some higher SES parents. These packages are obtained from a local farmer or organization. The preference for this local aspect for both healthy and sustainable food choices was mostly because of the trust in small local businesses, such as a nearby farmer, reducing over choice in stores, receiving new cooking inspiration and tasty seasonal vegetables. However different reasons were mentioned against food packages, such as the online ordering system, the lack of flexible pick-up timings and points, the lack of their geographical availability, and the higher perceived price and less varied content.

*“You don’t know what you get in advance, but that makes it fun, and also exciting for the children.” [P*_*high*_* F, 30yrs]*

#### Inspiration via (social) media

Media sources for inspiration to cook healthy and sustainable are frequently consulted by both groups of SES parents. This way, cookbooks and magazines are inspiring offline sources, as well as online magazines, web searches (e.g. through Google) and television programs. Role models seem to be reliable sources for both SES groups. Furthermore, many higher SES parents appreciated seasonal calendars of vegetables and fruits to make healthy and sustainable food choices, whereas lower SES parents mostly mentioned using television shows, internet sites, YouTube or social media in gathering information.

*“I look on YouTube for inspiration, for a program with a Brazilian girl. (…) I try to do what she does” [P*_*low*_* F, 42yrs]*

*“Yes I have a folder with all my favorite dishes and before I go to the store I take a look at it and select my recipes.” [P*_*high*_* F, 48yrs]*

#### Other mentioned intervention strategies

Some higher SES parents referred to the role of the government to promote more healthy eating, by for example subsidizing the purchase of healthy foods. There should also be more awareness-raising campaigns to stipulate the importance of sustainable foods.

A specific project installed by a Belgian supermarket chain together with social organizations, was discussed with lower SES participants. The project offers them two-weekly booklets with recipes of healthy and sustainable meals, and the ingredients can be bought at a reduced price. The price reduction is given automatically by scanning a loyalty card at the cashier. In the discussions most lower SES parents indicated to be interested in this project because of the inspiration it brings, the possibility to try varied meals and the low prices.

## Discussion

**Fig. 1:**
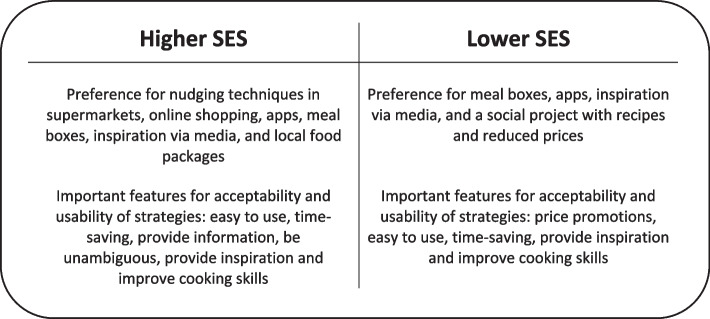
Main conclusions for higher and lower SES parents

This study gained insight into the preference for, and the usability and acceptability of various intervention strategies to promote healthy and sustainable food choices. As expected, we found differences between lower and higher SES parents. Figure 1 gives an overview of the main conclusions.

First of all, many higher SES parents spontaneously came up with examples and ideas for potential helpful intervention strategies, whereas lower SES parents found the question difficult to answer and needed more direction. Second, most higher SES parents already knew the strategies or tried them out, while lower SES parents did not (i.e., online shopping, meal boxes, the Nutri-score label). Even though lower SES parents had not used these strategies up until then, we still explored their interest to try them out, and meanwhile assessed the strategies’ usability and acceptability. Hence, perceived advantages and barriers for both SES groups became clear. Furthermore, alleged usability, acceptability, and preference of intervention strategies was generally similar for healthy as well as sustainable food choices. Both parent groups indicated that a distinction in strategies to promote healthy or sustainable food choices is not necessary, suggesting that perceived advantages and disadvantages of a strategy are the same for both types of food choices.

Since nudges influence consumers mostly in an unconscious way [41], parents’ conscious views on these nudges’ effectiveness can differ from the actual impact on their buying behavior. In this qualitative study most higher SES parents were in favor of visibility and convenience enhancements (e.g. placing healthy and sustainable products at eye level, providing more healthy and sustainable pre-cooked meals) in the grocery store. Research shows that nudges improving the visibility and convenience of healthy foods are approved of [42] but are not necessarily the most popular ones [42, 43]. Most lower SES parents did not believe in, or showed interest in these nudging techniques. Nevertheless, a recent systematic review showed that disadvantaged people would benefit more from nudges that demand less agency and are more behaviorally oriented [44], confirming the importance of structural interventions for lower SES people [22]. In our study higher SES parents were familiar with the Nutri-Score label, while most lower SES parents were not. This unawareness of the label might be explained by the importance lower SES parents attach to the price of foods (e.g. looking for promotions), more than the healthfulness or sustainability of the products [45], as well as by the grocery stores they shop in. Lower SES parents regularly shop for groceries in discount supermarkets, where the Nutri-score is rarely used. Surprisingly, a few lower SES parents preferred looking at descriptive labels and separate ingredients instead of evaluative labels (i.e.; the Nutri-Score), while previous research showed that people with lower SES showed a good comprehension of evaluative labels [46] and are generally less likely to comprehend descriptive nutrition labels [47].

As is the case in most industrialized countries, also in Belgium, food is increasingly bought online (i.e., grocery store purchases, prepared food delivered at home and meal boxes delivered at home) [48]. About half of the higher SES parents stated to shop for groceries online, appreciating saving time and reducing temptation. This perceived advantage is confirmed by Huyghe et al. [49] who found that consumers are likely to buy fewer unhealthy products in an online than in an offline shopping environment. Moreover, parents suggested a clearly visible healthy and sustainable label to guide them in their online purchases, which is in accordance with a recent study that shows that Nutri-Score labeling in an online store environment can guide consumers toward healthier food choices [50]. Regarding apps, a literature review of Coughlin et al. [51] found similar results to our findings about the preference of applications being quick and easy. Half of the lower and higher SES parents were interested in apps. They mentioned that features should provide a filter function for healthy and sustainable products, recipes with ingredients and a shopping list. Considering the increasing tendency of purchasing food online, it is relevant that our results point to a certain readiness to adopt e- and mHealth tools, at least among higher SES parents. Lower SES parents did not show interest in online shopping interventions, but did so in the use of applications. Nevertheless, lower SES parents stated never to use an application for grocery shopping. This might be explained by prioritizing the price of products, and the fact that most smartphone or web applications are usually designed for more affluent people [52, 53]. People with lower SES often tend to have lower eHealth literacy [54], making digital interventions to be less effective in lower SES populations [55], and possibly even widening social health inequalities [52].

As expected [34, 48] both parent groups in our study appreciated the time-saving aspect of meal boxes delivered at home. However, some higher SES parents also mentioned that the preparation of the recipes was rather time consuming. Not only the convenience of meal boxes was appreciated by both parent groups, also the opportunity to improve cooking skills, and the inspiration they provide to try new dishes (e.g., vegetarian meals), was valued. Even though the use of meal boxes seems to have a positive effect on children (e.g., engaging children in cooking, willingness to try foods) [56], in our study children seemed to be a barrier to use meal boxes. Some higher SES parents indicated preparing a different meal for their children when using a meal box for themselves, whilst lower SES parents showed concern about whether their children would eat the meals, so to avoid the risk of wasting money. This is an important barrier to overcome when developing an intervention study with meal box schemes that aims to improve children’s dietary habits. Interestingly, lower SES parents never used the meal boxes before but would like to have the opportunity to do so, if the obstacle of the high price could be removed. This reported interest and a small but significant positive effect on dietary habits of children in lower SES families found by earlier research [36, 37, 57], makes home-delivered meal boxes a promising practice to improve families’ food choices. Moreover, one lower SES parent mentioned interest in the project of a Belgian supermarket chain together with social organizations, that offers people of lower SES booklets including recipes of healthy and sustainable meals that cost very little (i.e., one, two or three euros per person). When questioning other lower SES parents about this project, most wanted to subscribe because of the reduced price and the inspiration it provides. The extra advantage of the reduced price of foods, and the liberty parents still have to buy other products, makes this project very promising as a way to help lower SES families making healthy and sustainable food choices.

In interpreting the results, some limitations should be considered. First, our study was conducted during the Covid-19 pandemic, which made it more difficult to recruit participants of lower SES and explains the discrepancy in the number of higher and lower SES participants. However, data saturation was reached in both parent groups since no new insights or themes emerged in the last focus group and individual interviews. Next, the order in which the questions were asked (i.e., first healthy, then sustainable food choices), generated more answers for interventions for healthy food choices. Parents’ ideas about interventions for sustainable food choices were mostly the same, suggesting that one intervention for both types of food choices could be interesting. Lastly, more directed questions about specific intervention strategies were used, possibly influencing the participant to provide a particular response, and biasing the results in a particular direction. Still, these questions provided insight in the acceptability and usability of less familiar intervention strategies. Strengths of the study include the diverse views from participants of varying SES. Moreover, our study is the first to examine the usability and acceptability of a range of helpful strategies to make healthy and sustainable food choices, while exploring differences between lower and higher SES parents. Due to the explorative research design, we gathered a lot of data and detailed insights in the topic.

## Conclusions and recommendations

Lower and higher SES parents showed interest in strategies to make both healthy and sustainable food choices easier. Strategies should be convenient, time saving, easy to use and provide inspiration for family meals. Moreover, children have an influence on parents’ food choices and should be considered when developing an intervention strategy. We found that when parents were restricted in choosing their own products, as is the case with a meal box or food package, children form a barrier to use the strategy since some lower and higher SES parents choose to adapt meals to what the children like. Despite similar perspectives of lower and higher SES parents on intervention strategies, findings also indicate differences between both groups. In contrast with lower SES parents, higher SES parents showed interest in an intervention via online shopping platforms, in visibility and convenience enhancements in the grocery store, and in fruit and vegetable packages from a local farmer. We could conclude that higher SES parents benefit from interventions that reduce choice stress (e.g., a product bundle), save time (e.g., home delivery), are easy to use (e.g., online platform with recipe ingredients linked to products that can easily be added to shopping carts or shopping lists), are unambiguous and provide information (e.g., one clear food label), provide inspiration and improve cooking skills (e.g., recipes to follow in a meal box or via different media sources). Compared to higher SES parents, lower SES parents also showed interest in a project that offers recipe booklets and ingredients at reduced price. For almost all lower SES parents, the price of food is the most important determinant in making food choices, regardless of other aspects of the intervention. Therefore, a structural intervention, such as loyalty cards that provide lower SES automatic discounts, might be promising for this population group [58, 59]. However, recent research takes it a step further and highlights the importance of a systems approach in health and sustainability promotion, stating that the impact of specific interventions depends on and interacts with other aspects of the food system, for example not only offering free fruits and vegetables packages to lower SES households, but also including them in a food community to increase exposure to healthy foods [60, 61]. Given that the impact of an intervention depends on how usable and acceptable it is for the receivers, our findings are an interesting added value for the development of a family-based intervention, as it provides a deeper understanding of what could help lower and higher SES parents make more healthy and sustainable food choices.

## Data Availability

The datasets analyzed during the current study are not publicly available due to privacy reasons, but are available from the corresponding author on reasonable request.

## Abbreviations

SES: Socioeconomic status
P_high_: Participant of higher SES
P_low_: Participant of lower SES
F: Female
yrs: Years

## References

[1] Tilman D, Clark M (2014). Global diets link environmental sustainability and human health. Nature.

[2] Aleksandrowicz L, Green R, Joy EJM, Smith P, Haines A (2016). The impacts of dietary change on greenhouse gas emissions, land use, water use, and health: a systematic review. PLoS One.

[3] Alimoradi Z, Golboni F, Griffiths MD, Broström A, Lin CY, Pakpour AH (2020). Weight-related stigma and psychological distress: a systematic review and meta-analysis. Clin Nutr.

[4] World Health Organization. Obesity and overweight. 2020. Available from: https://www.who.int/news-room/fact-sheets/detail/obesity-and-overweight. [Cited 2021 May 26].

[5] Willett (2019). Food in the Anthropocene: the EAT–Lancet Commission on healthy diets from sustainable food systems. Lancet.

[6] Song M, Fung T, Hu F, Willett W, Longo V, Chan A (2016). Association of animal and plant protein intake with all-cause and cause-specific mortality. JAMA Intern Med.

[7] Nelson ME, Hamm MW, Hu FB, Abrams SA, Griffin TS (2016). Alignment of healthy dietary patterns and environmental sustainability: a systematic review. Adv Nutr.

[8] Moore O, Wilkie E, Desrochers W (2016). All in the family? Parental roles in the epidemic of childhood obesity. J Consum Res.

[9] Nicklaus S, Boggio V, Chabanet C, Issanchou S (2004). A prospective study of food preferences in childhood. Food Qual Prefer.

[10] Nu CT, MacLeod P, Barthelemy J (1996). Effects of age and gender on adolescents’ food habits and preferences. Food Qual Prefer.

[11] Vaughn AE, Ward DS, Fisher JO, Faith MS, Hughes SO, Kremers SPJ (2016). Fundamental constructs in food parenting practices: a content map to guide future research. Nutr Rev.

[12] Yee AZH, Lwin MO, Ho SS (2017). The influence of parental practices on child promotive and preventive food consumption behaviors: a systematic review and meta-analysis. Int J Behav Nutr Phys Act.

[13] Zarnowiecki D, Ball K, Parletta N, Dollman J (2014). Describing socioeconomic gradients in children’s diets - does the socioeconomic indicator used matter?. Int J Behav Nutr Phys Act.

[14] Giskes K, Avendaňo M, Brug J, Kunst AE (2010). A systematic review of studies on socioeconomic inequalities in dietary intakes associated with weight gain and overweight/obesity conducted among European adults. Obes Rev.

[15] Zarnowiecki DM, Dollman J, Parletta N (2014). Associations between predictors of children’s dietary intake and socioeconomic position: a systematic review of the literature. Obes Rev.

[16] Correia D, Severo M, Lopes C (2020). The role of socio-economic factors in food consumption of Portuguese children and adolescents: Results from the National Food, Nutrition and Physical Activity Survey 2015–2016. Br J Nutr.

[17] Ranjit N, Wilkinson A V., Lytle LM, Evans AE, Saxton D, Hoelscher DM. Socioeconomic inequalities in children’s diet: The role of the home food environment. Int J Behav Nutr Phys Act. 2015;12(1):S4. 10.1186/1479-5868-12-S1-S4 PMC4518619 26222785

[18] Chung A, Backholer K, Wong E, Palermo C, Keating C, Peeters A (2016). Trends in child and adolescent obesity prevalence in economically advanced countries according to socioeconomic position: a systematic review. Obes Rev.

[19] Beauchamp A, Backholer K, Magliano D, Peeters A (2014). The effect of obesity prevention interventions according to socioeconomic position: a systematic review. Obes Rev.

[20] McGill R, Anwar E, Orton L, Bromley H, Lloyd-Williams F, O’Flaherty M (2015). Are interventions to promote healthy eating equally effective for all? Systematic review of socioeconomic inequalities in impact Health behavior, health promotion and society. BMC Public Health.

[21] Lorenc T, Petticrew M, Welch V, Tugwell P (2013). What types of interventions generate inequalities? Evidence from systematic reviews. J Epidemiol Community Health.

[22] Adams J, Mytton O, White M, Monsivais P (2016). Why are some population interventions for diet and obesity more equitable and effective than others? The role of individual agency. PLoS Med.

[23] Campbell S, James EL, Stacey FG, Bowman J, Chapman K, Kelly B (2014). A mixed-method examination of food marketing directed towards children in Australian supermarkets. Health Promot Int.

[24] Lawson GM, Hook CJ, Farah MJ (2018). A meta-analysis of the relationship between socioeconomic status and executive function performance among children. Dev Sci.

[25] Frieden TR (2010). A framework for public health action: The health impact pyramid. Am J Public Health.

[26] Sekhon M, Cartwright M, Francis JJ (2017). Acceptability of healthcare interventions: an overview of reviews and development of a theoretical framework. BMC Health Serv Res.

[27] Vos M, Deforche B, van Kerckhove A, Michels N, Poelman M, Geuens M, et al. Determinants of healthy and sustainable food choices in parents with a higher and lower socioeconomic status: A qualitative study. Appetite. 2022 Nov 1;178:106180. Available from: https://linkinghub.elsevier.com/retrieve/pii/S0195666322002719. [Cited 2022 Aug 29] 10.1016/j.appet.2022.106180 35863506

[28] Khan SA, Sowards SK (2018). It’s Not Just Dinner: Meal Delivery Kits as Food Media for Food Citizens. Front Commun (Lausanne).

[29] Fitzgerald A, Heary C, Nixon E, Kelly C (2010). Factors influencing the food choices of Irish children and adolescents: a qualitative investigation. Health Promot Int.

[30] Gezond Leven VI. Voedingsdriehoek | Gezond Leven. 2017. Available from: https://www.gezondleven.be/themas/voeding/voedingsdriehoek. [Cited 2021 Apr 5].

[31] Broers VJV, De Breucker C, Van Den Broucke S, Luminet O (2017). A systematic review and meta-analysis of the effectiveness of nudging to increase fruit and vegetable choice. Eur J Public Health.

[32] Cecchini M, Warin L (2016). Impact of food labelling systems on food choices and eating behaviours: a systematic review and meta-analysis of randomized studies. Obes Rev.

[33] Hollands GJ, Carter P, Anwer S, King SE, Jebb SA, Ogilvie D (2019). Altering the availability or proximity of food, alcohol, and tobacco products to change their selection and consumption. Cochrane Database Syst Rev.

[34] Hertz FD, Halkier B (2017). Meal box schemes a convenient way to avoid convenience food? Uses and understandings of meal box schemes among Danish consumers. Appetite.

[35] Flaherty SJ, McCarthy M, Collins A, McAuliffe F (2018). Can existing mobile apps support healthier food purchasing behaviour? Content analysis of nutrition content, behaviour change theory and user quality integration. Public Health Nutr.

[36] Utter J, Denny S (2016). Supporting families to cook at home and eat together: findings from a feasibility study. J Nutr Educ Behav.

[37] Cabili C, Briefel R, Forrestal S, Gabor V, Chojnacki G (2021). A cluster randomized controlled trial of a home-delivered food box on children’s diet quality in the Chickasaw nation packed promise project. J Acad Nutr Diet.

[38] Braun V, Clarke V (2006). Using thematic analysis in psychology. Qual Res Psychol.

[39] Reynders T, Nicaise I, Van Damme J. De constructie van een SES-variabele voor het SiBO-onderzoek. Vol. 31, LOA-rapport. 2005.

[40] Volksgezondheid F. Nutri-Score | FOD Volksgezondheid. 2019. Available from: https://www.health.belgium.be/nl/de-nutri-score-0. [Cited 2022 Feb 4].

[41] Thaler R, Sunstein CR (2009). Nudge: improving decisions about health, wealth, and happiness. Penguin Books.

[42] Van Gestel LC, Kroese FM, De Ridder DTD (2018). Nudging at the checkout counter – A longitudinal study of the effect of a food repositioning nudge on healthy food choice. Psychol Health.

[43] Jilcott Pitts SB, Wu Q, Sharpe PA, Rafferty AP, Elbel B, Ammerman AS (2016). Preferred healthy food nudges, food store environments, and customer dietary practices in 2 low-income Southern Communities. J Nutr Educ Behav.

[44] Schüz B, Meyerhof H, Hilz LK, Mata J. Equity effects of dietary nudging field experiments: systematic review. Front Public Health. 2021;23(9):668998. 10.3389/fpubh.2021.668998 PMC8342848 34368049

[45] Daniel C (2016). Economic constraints on taste formation and the true cost of healthy eating. Soc Sci Med.

[46] Egnell M, Talati Z, Galan P, Andreeva VA, Vandevijvere S, Gombaud M (2020). Objective understanding of the Nutri-score front-of-pack label by European consumers and its effect on food choices: an online experimental study. Int J Behav Nutr Phys Act.

[47] Sinclair S, Hammond D, Goodman S (2013). Sociodemographic differences in the comprehension of nutritional labels on food products. J Nutr Educ Behav.

[48] Comeos, Pieter De Vuyst, Leen Boels IP. E-commerce Belgium 2019. Available from: https://static.comeos.be/E-commerce_Belgium_2019__2.pdf. [Cited 2021 Apr 15].

[49] Huyghe E, Verstraeten J, Geuens M, Van Kerckhove A (2017). Clicks as a Healthy Alternative to Bricks: How Online Grocery Shopping Reduces Vice Purchases. J Mark Res.

[50] Jansen L, van Kleef E, Van Loo EJ (2021). The use of food swaps to encourage healthier online food choices: a randomized controlled trial. Int J Behav Nutr Phys Act.

[51] Coughlin SS, Whitehead M, Sheats JQ, Mastromonico J, Hardy D, Smith SA (2015). Smartphone applications for promoting healthy diet and nutrition: a literature review. Jacobs J Food Nutr.

[52] Latulippe K, Hamel C, Giroux D (2017). Social health inequalities and eHealth: a literature review with qualitative synthesis of theoretical and empirical studies. J Med Internet Res.

[53] Mackert M, Mabry-Flynn A, Champlin S, Donovan EE, Pounders K (2016). Health Literacy and Health Information Technology Adoption: The Potential for a New Digital Divide. J Med Internet Res.

[54] Neter E, Brainin E (2012). eHealth literacy: extending the digital divide to the realm of health information. J Med Internet Res.

[55] Western MJ, Armstrong MEG, Islam I, Morgan K, Jones UF, Kelson MJ (2021). The effectiveness of digital interventions for increasing physical activity in individuals of low socioeconomic status: a systematic review and meta-analysis. Int J Behav Nutr Phys Act.

[56] Fraser K, Love P, Campbell KJ, Ball K, Opie RS (2022). Meal kits in the family setting: Impacts on family dynamics, nutrition, social and mental health. Appetite.

[57] Zeldman J, Mialki K, Sweeney L, Shelnutt K (2020). O27 family mealtime behaviors among low-income African Americans participating in a healthy meal kit intervention. J Nutr Educ Behav.

[58] Black AP, Brimblecombe J, Eyles H, Morris P, Vally H, Dea KO (2012). Food subsidy programs and the health and nutritional status of disadvantaged families in high income countries: a systematic review. BMC Public Health.

[59] Afshin A, Peñalvo JL, Del Gobbo L, Silva J, Michaelson M, O’Flaherty M (2017). The prospective impact of food pricing on improving dietary consumption: a systematic review and meta-analysis. PLoS One.

[60] Friel S, Pescud M, Malbon E, Lee A, Carter R, Greenfield J, et al. Using systems science to understand the determinants of inequities in healthy eating. Bammann K, editor. PLoS One 2017;12(11):e0188872. 10.1371/journal.pone.0188872 10.1371/journal.pone.0188872 PMC5708780 29190662

[61] Sawyer ADM, van Lenthe F, Kamphuis CBM, Terragni L, Roos G, Poelman MP (2021). Dynamics of the complex food environment underlying dietary intake in low-income groups: a systems map of associations extracted from a systematic umbrella literature review. Int J Behav Nutr Phys Act.

